# HbA1c as a shared treatment goal in type 2 diabetes? A secondary analysis of the DEBATE trial

**DOI:** 10.1186/s12875-023-02067-9

**Published:** 2023-05-13

**Authors:** Sara Santos, Michael Pentzek, Attila Altiner, Anne Daubmann, Eva Drewelow, Christian Helbig, Christin Löffler, Susanne Löscher, Karl Wegscheider, Heinz-Harald Abholz, Stefan Wilm, Anja Wollny

**Affiliations:** 1 Institute of General Practice (ifam), Medical Faculty, Centre for Health & Society (chs), Heinrich Heine University Düsseldorf, Moorenstr. 5, 40225 Düsseldorf, Germany; 2 Institute of General Practice and Primary Care, Chair of General Practice II and Patient Centredness in Primary Care, Faculty of Health/School of Medicine, Witten/Herdecke University, Alfred-Herrhausen-Straße 50, 58448 Witten, Germany; 3 Department of General Practice and Health Services Research, Heidelberg University Hospital, Im Neuenheimer Feld 130.3, 69120 Heidelberg, Germany; 4 Institute of General Practice, Rostock University Medical Center, Doberaner Str. 142, 18057 Rostock, Germany; 5 Institute of Medical Biometry and Epidemiology, University Medical Center Hamburg-Eppendorf, Martinistraße 52, 20246 Hamburg, Germany

**Keywords:** Diabetes mellitus type 2, Decision making, General Practice, Glycated hemoglobin A

## Abstract

**Background:**

Type 2 diabetes mellitus (T2DM) is a major health problem in the western world. Despite a widespread implementation of integrated care programs there are still patients with poorly controlled T2DM. Shared goal setting within the process of Shared Decision Making (SDM) may increase patient’s compliance and adherence to treatment regimen. In our secondary analysis of the cluster-randomized controlled DEBATE trial, we investigated if patients with shared vs. non-shared HbA1c treatment goal, achieve their glycemic goals.

**Methods:**

In a German primary care setting, we collected data before intervention at baseline, 6, 12 and 24 months. Patients with T2DM with an HbA1c ≥ 8.0% (64 mmol/mol) at the time of recruitment and complete data at baseline and after 24 months were eligible for the presented analyses. Using a generalized estimating equation analysis, we analysed the association between the achievement of HbA1c goals at 24 months based on their shared vs. non-shared status, age, sex, education, partner status, controlled for baseline HbA1c and insulin therapy.

**Results:**

From N = 833 recruited patients at baseline, n = 547 (65.7%) from 105 General Practitioners (GPs) were analysed. 53.4% patients were male, 33.1% without a partner, 64.4% had a low educational level, mean age was 64.6 (SD 10.6), 60.7% took insulin at baseline, mean baseline HbA1c was 9.1 (SD 1.0). For 287 patients (52.5%), the GPs reported to use HbA1c as a shared goal, for 260 patients (47.5%) as a non-shared goal. 235 patients (43.0%) reached the HbA1c goal after two years, 312 patients (57.0%) missed it. Multivariable analysis shows that shared vs. non-shared HbA1c goal setting, age, sex, and education are not associated with the achievement of the HbA1c goal. However, patients living without a partner show a higher risk of missing the goal (p = .003; OR 1.89; 95% CI 1.25–2.86).

**Conclusions:**

Shared goal setting with T2DM patients targeting on HbA1c-levels had no significant impact on goal achievement. It may be assumed, that shared goal setting on patient-related clinical outcomes within the process of SDM has not been fully captured yet.

**Trial registration:**

The trial was registered at ISRCTN registry under the reference ISRCTN70713571.

**Supplementary Information:**

The online version contains supplementary material available at 10.1186/s12875-023-02067-9.

## Background

Diabetes is one of the most common chronic disorders and a major public health problem in the western world which leads to an enormous economic burden; type 2 diabetes mellitus (T2DM) constitutes about 85–95% of all diabetes cases [1, 2]. In Germany the diabetes prevalence increased from 4.7 to 5.6% in the past two decades [1].

Recent preventive therapeutic interventions and treatment concepts have been focusing to reduce and avoid microvascular and macrovascular complications, such as vision loss, cardiovascular disease, and kidney failure [3–7]. Despite of a widespread implementation of integrated care programs [8] there is still a group of patients with diabetes, who do not achieve treatment recommendations [9]. Among patients with T2DM registered in the German Disease Management Programme (DMP), 10% show poor control [10].

In Germany T2DM patients are primarily treated in general practice [11]. A standard for assessing long-term blood glucose control is the measurement of glycated hemoglobin (HbA1c), as it is a surrogate marker for longterm complications [12, 13].

Former guidelines recommended HbA1c levels of less than 7%, however most patients did not achieve these recommended goals [3, 14]. This may be due to the fact that these goals are neither realistic nor desirable in heterogeneous populations such as patients at increased risk of hyperglycemia or patients with limited life expectancy [15, 16]. Therefore the German National Disease Management Guideline on treatment of T2DM had abandoned the use of a single glycemic goal for all patients and favour individualized treatment goals including HbA1c values between 6.5 and 8.5% [17].

Setting individualized treatment goals tailored to patients needs and preferences may reduce the risk for diabetic complications and help patients achieve their treatment targets [18, 19].

As setting individualized treatment goals needs a participatory approach to be more effective, it seems reasonable to incorporate goal setting in the concept of Shared Decision Making (SDM) [20, 21]. SDM is defined as a decision-making process jointly shared between patients and their healthcare providers [22, 23]. The goal of this concept is to come to a jointly responsible agreement, that takes into account patient preferences and competencies, including evidence-based information. SDM can therefore make an important contribution to aligning HbA1c targets with patient values [24, 25].

In previous studies we could show how important the HbA1c monitoring for GPs in type 2 diabetes management is [26–28].

In this secondary analysis of the DEBATE trial [26] we aim to describe the HbA1c goals of the participants of the trial, identify differences in the achievement of shared versus non-shared HbA1c goals and their relationship to diabetes outcomes.

## Methods

The presented secondary analyses are based on data of the cluster-randomized controlled DEBATE trial [26], which examined the impact of patient-centered communication and SDM between GPs and their patients with poorly controlled T2DM on improving blood glucose levels. The intervention concept was composed of (1) peer-visits (GPs specially trained in patient-centered communication and who were members of the study team visited enrolled GPs) with the aim to sensitize patients to their disease concepts and their views, attitudes and behaviors by using patient-centered communication. And (2) the use of an electronic decision-aid. GPs were encouraged to use the electronic decision-aid (https://www.arriba-hausarzt.de/) to increase SDM.

Data was measured before randomization (baseline, T0), at 6–8 (T1), 12–14 (T2), 18–20 (T3) and 24–26 months (T4) after baseline. The DEBATE trial was not able to show a significant difference on the primary outcome (level of HbA1c) between intervention and control group. A detailed description of the intervention, methods and results of the DEBATE trial are published elsewhere [26, 29]. We recruited GPs registered in regional Associations of Statutory Health Insurance Physicians of the German regions Mecklenburg-Western Pomerania and North Rhine-Westphalia. Each participating GP practice compiled a list of eligible patients, which were contacted and included, when they fulfilled the inclusion criteria: being affected by T2DM and having an HbA1c level of ≥ 8.0% (64 mmol/mol) within three months prior to recruitment. Detailed information on the process of recruitment have been published elsewhere [29].

### Measurement tools

Detailed information on data collection procedures in the DEBATE trial were published elsewhere [29].

#### Patient questionnaire

Sociodemographic data (sex, age, partner status with/without, education ≤8 years/>8 years) and psychological data (see below) were collected via telephone interviews by study team members at the different time points of measurement.

The SDM-Q-9 (Shared Decision Making questionnaire; 9 items scored 0–5, total score is the sum 0–45, more SDM with higher score) measures patient-perceived involvement in medical decision making [30].

The PACIC (patient assessment of chronic illness care; 11 items scored 1–5, score is the mean 1–5, better care with higher score) measures patient-perceived patient-centeredness in chronic care [31].

The PAID (problem areas in diabetes; 20 items scored 0–4, total score is the sum multiplied by 1.25 = 0-100, more distress with higher score) measures diabetes-related emotional distress [32].

The EQ-5D-3 L (European quality of life 5 dimensions 3 level version; 5 items with 3 levels, total score is the weighted EQ-5D index from low negative values to 1.0, better quality of life with higher score) measures quality of life [33].

#### GP questionnaire including treatment goals

GPs gave information about their participating patients on a questionnaire (age, sex, measured HbA1c, pharmacotherapy).

At T0 GPs indicated once (a) which HbA1c target value they have set together with their patients (shared treatment goal) and (b) if no shared target value has been set, what target value they would like to see for their patients (non-shared treatment goal).

At T2 and T4, only the measured HbA1c value was collected.

It was not investigated which factors GPs used for the decision to escalate medication and whether escalating medication had an impact on achieving the shared HbA1c goals.

### Statistical methods

Patients with T2DM with an HbA1c ≥ 8.0% (64 mmol/mol) at the time of recruitment and complete data at baseline and T4 were eligible for the presented analyses (see Additional file 1).

For a description of the sample, we compared the subgroups of patients with shared HbA1c goal and with non-shared HbA1c goal, using t tests and χ² tests for independent samples. For a description of the magnitude of differences, we calculated Cohen’s d as an effect size from the T statistic and the phi coefficient, respectively [34]. We use a common interpretation of Cohen’s d: <0.20 no/minimal, 0.20–0.49 small, 0.50–0.80 medium, > 0.80 large effect [35].

For the main analysis, we calculated a multivariable generalized estimating equation (GEE), adjusting for dependencies between patient-level data and affiliation with a specific GP. The criterion was “HbA1c goal missed vs. achieved at T4” and the baseline predictors were “HbA1c as shared vs. non-shared goal”, age, sex, education, partner status, controlled for baseline HbA1c and insulin therapy. We defined an HbA1c goal as “missed” if the measured HbA1c at T4 was > 0.5 above the aspired HbA1c goal at baseline. Research has shown, that a HbA1c reduction of 0.5% is clinically relevant and leads to less absenteeism, diabetes-related hospital admissions and improved quality of life [36, 37].

Sensitivity analyses for this main analysis were performed with regard to (1) including the intervention of the DEBATE trial (control group vs. intervention group) [29] as an additional predictor variable, (2) a metric criterion (difference between measured HbA1c at T4 and HbA1c goal) instead of a dichotomous criterion (missed vs. achieved goal), (3) using outcome criteria (missed/achieved goal and difference between measured and aspired HbA1c, respectively) from T2 (1 year after baseline) instead of T4, (4) including the shared-decision making score (SDM-Q-9) as an additional predictor variable (as a main effect and as an interaction term SDM x shared/non shared HbA1c goal). Results of the sensitivity analyses are shown in Additional file 2: sensitivity analyses.

A secondary question is whether the type of HbA1c goal setting (shared vs. non-shared) is associated not only with goal achievement (main analysis), but also with the overall diabetes outcome. We therefore built a composite score for HbA1c, quality of life, and diabetic distress from z-standardized HbA1c, EQ-5D-3 L, and PAID, both for baseline and T4 (higher score = worse outcome (higher HbA1c/more distress/lower QoL)). We performed a GEE with the T4 composite score as the criterion, the same predictors mentioned above, and controlled for the baseline composite score.

The significance level was 0.05. Analyses were performed with SPSS (IBM Corp. Released 2017. IBM SPSS Statistics for Windows, Version 25.0. Armonk, NY: IBM Corp.).

## Results

### Recruitment and participant flow

All patients with an HbA1c value of ≥8.0 at baseline and complete values at T0 and T4 for the primary analyses (decision shared/non-shared, socio-demographics, HbA1c goal, measured HbA1c, insulin vs. no insulin) were included (n = 547; see Additional file 1: flow chart).

### Baseline data

GPs state a shared HbA1c goal for n = 287 patients (52.47%) and a non-shared HbA1c goal for n = 260 patients (47.53%). Patients with shared HbA1c goal show slightly lower HbA1c values (both measured and aspired) than patients with non-shared HbA1c goal (see Table 1 for all comparisons).

**Table 1:** Baseline (T0) characteristics including bivariate comparisons of the samples shared vs. non-shared HbA1c goal

Parameter		Total (N = 547)	Sample with **shared** HbA1c goal (n = 287; 52.47%)	Sample with **non**-shared HbA1c goal (n = 260; 47.53%)	p	Effect size (Cohen’s d)
Age T0, mean (SD)		64.61 (10.63)	64.48 (10.64)	64.75 (10.63)	0.762	0.026
Sex, n (%)	women	255 (46.62)	145 (50.52)	110 (42.31)	0.054	0.165
	men	292 (53.38)	142 (49.48)	150 (57.69)		
Education, n (%)	≤8 years	352 (64.35)	178 (62.02)	174 (66.92)	0.232	0.102
	> 8 years	195 (35.65)	109 (37.98)	86 (33.08)		
Partner status, n (%)	with	366 (66.91)	187 (65.16)	179 (68.85)	0.360	0.078
	without	181 (33.09)	100 (34.84)	81 (31.15)		
HbA1c goal, mean (SD)		7.46 (0.58)	7.36 (0.57)	7.57 (0.56)	< 0.001	**0.380**
HbA1c measured at T0, mean (SD)		9.10 (1.04)	9.00 (0.97)	9.22 (1.10)	0.018	**0.204**
Difference between measured HbA1c (T0) and HbA1c goal, mean (SD)		1.64 (1.05)	1.65 (1.00)	1.64 (1.11)	0.964	0.004
Insulin treatment T0, n (%)	yes	332 (60.69)	164 (57.14)	168 (64.62)	0.074	0.153
	no	215 (39.31)	123 (42.86)	92 (35.38)		
SDM-Q-9 T0 (n = 9 missing), mean (SD)		24.40 (14.05)	24.21 (14.43)	24.62 (13.62)	0.736	0.029
PACIC T0 (n = 2 missing), mean (SD)		2.44 (0.84)	2.47 (0.80)	2.41 (0.89)	0.414	0.070
PAID T0 (n = 9 missing), mean (SD)		14.22 (15.02)	14.25 (14.80)	14.20 (15.30)	0.970	0.003
EQ-5D-3 L index T0 (n = 3 missing), mean (SD)		0.67 (0.31)	0.66 (0.32)	0.68 (0.31)	0.470	0.062

HbA1c glycated haemoglobin; SD standard deviation; SDM-Q-9 shared decision making (9 items scored 0–5, total score is the sum 0–45, more SDM with higher score); PACIC patient assessment of chronic illness care (11 items scored 1–5, score is the mean 1–5, better care with higher score); PAID problem areas in diabetes (20 items scored 0–4, total score is the sum multiplied by 1.25 = 0-100, more distress with higher score); EQ-5D-3 L quality of life (5 items with 3 levels, total score is the weighted EQ-5D index from low negative values to 1.0, better QoL with higher score)

**Fig. 1:**
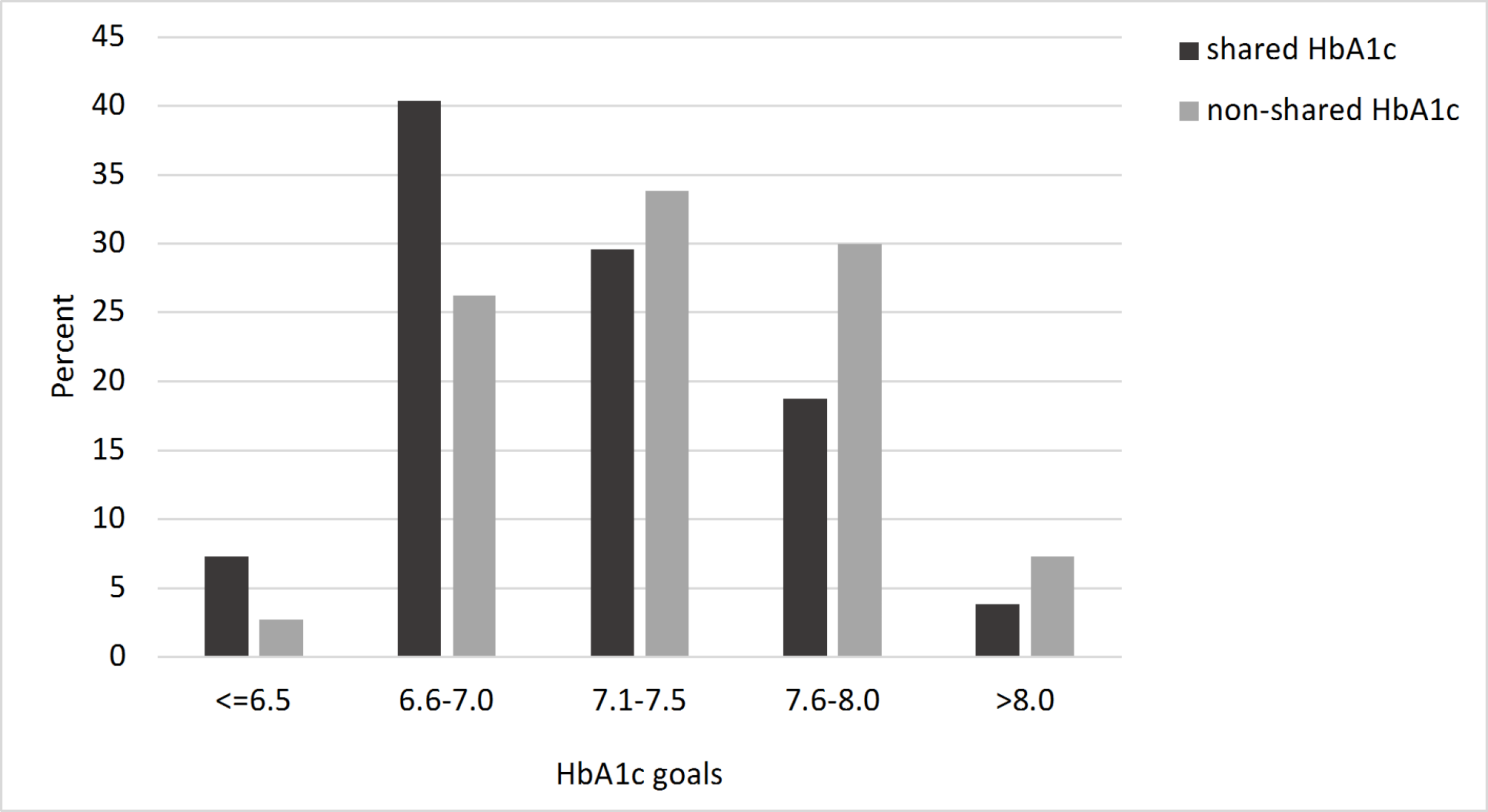
Frequency of GP-reported shared vs. non-shared HbA1c goals (N = 547)

In addition to the metric comparison in Table 1; Fig. 1 also shows the frequency distribution of various clinically relevant HbA1c goal categories between the two groups: The HbA1c is used more often as a shared goal with lower (stricter) aspired HbA1c, whereas non-shared HbA1c usage is associated with a higher (more liberal) HbA1c goal (Pearson’s χ² (df = 4) = 24.798; p < .001).

### Longitudinal outcomes

After two years (T4), 235 (43%) achieved the HbA1c goal, and 312 (57%) missed the goal. As with baseline HbA1c, patients with a shared HbA1c goal also show slightly lower HbA1c at T4 (see Table 2 for all comparisons).

**Table 2:** Follow-up characteristics (T4) including bivariate comparisons of the samples shared vs. non-shared HbA1c goal

Parameter		Total (N = 547)	Sample with **shared** HbA1c goal (n = 287; 52.47%)	Sample with **non**-shared HbA1c goal (n = 260; 47.53%)	p	Effect size (Cohen’s d)
HbA1c measured at T4, mean (SD)		8.36 (1.42)	8.18 (1.28)	8.55 (1.53)	0.003	**0.260**
Difference between HbA1c goal and measured HbA1c (T4), mean (SD)		0.90 (1.46)	0.83 (1.30)	0.98 (1.61)	0.226	0.104
HbA1c goal achieved at T4, n (%)	yes	235 (42.96)	127 (44.25)	108 (41.54)	0.522	0.055
	no	312 (57.04)	160 (55.75)	152 (58.46)		
Insulin treatment T4, n (%)	yes	405 (74.04)	205 (71.43)	200 (76.92)	0.143	0.125
	no	142 (25.96)	82 (28.57)	60 (23.08)		
SDM-Q-9 T4 (n = 2 missing), mean (SD)		20.57 (14.79)	21.85 (14.73)	19.14 (14.76)	0.032	0.184
PACIC T4 (n = 5 missing), mean (SD)		2.53 (0.93)	2.52 (0.92)	2.54 (0.94)	0.767	0.025
PAID T4 (n = 9 missing), mean (SD)		12.95 (14.18)	13.10 (13.32)	12.80 (15.09)	0.806	0.021
EQ-5D-3 L index T4 (n = 1 missing), mean (SD)		0.67 (0.33)	0.68 (0.33)	0.67 (0.33)	0.967	0.004

HbA1c glycated haemoglobin; SD standard deviation; SDM-Q-9 shared decision making (9 items scored 0–5, total score is the sum 0–45, more SDM with higher score); PACIC patient assessment of chronic illness care (11 items scored 1–5, score is the mean 1–5, better care with higher score); PAID problem areas in diabetes (20 items scored 0–4, total score is the sum multiplied by 1.25 = 0-100, more distress with higher score); EQ-5D-3 L quality of life (5 items with 3 levels, total score is the weighted EQ-5D index from low negative values to 1.0, better QoL with higher score)

Multivariable analysis (Table 3) reveals that shared vs. non-shared use of an HbA1c goal is not associated with goal achievement. Age, sex and education are not associated, too. However, living without a partner and insulin medication are associated with a higher chance of missing the HbA1c goal two years later.

**Table 3:** Multivariable influences on outcome „HbA1c goal missed”*

Predictor	Wald’s χ²	df	p	Odds ratio	95% confidence interval
Shared HbA1c goal (vs. non-shared HbA1c goal)	0.130	1	0.718	0.934	0.646–1.351
Women (vs. men)	0.058	1	0.810	1.043	0.742–1.466
Being without partner (vs. with partner)	9.063	1	0.003	1.889	1.249–2.858
Education > 8 years (vs. ≤8 years)	1.553	1	0.213	1.292	0.864–1.933
Insulin (vs. No insulin)	3.966	1	0.046	1.475	1.006–2.163
HbA1c value T0	10.204	1	0.001	1.370	1.129–1.661
Age	1.578	1	0.209	0.989	0.972–1.006

Generalized Estimating Equation with cluster effect “GP practice”, binomial, logit link

*higher outcome = higher risk of missing the goal (measured HbA1c at T4 is > 0.5 above goal); T0 baseline, T4 two years after baseline

Sensitivity analyses are shown in Additional file 2 tables. The direction and pattern of results is robust: Shared vs. non-shared HbA1c use shows no association with goal achievement, whereas living with a partner and taking no insulin are associated with better goal achievement. With T2 criterion (1 year after baseline), higher age is also slightly associated with better goal achievement.

Concerning the overall diabetes outcome (composite score), shared vs. non-shared use of an HbA1c goal also shows no association (Table 4). In contrast to the main analysis partner status is not associated. Also sex shows no association, while lower age, taking no insulin and higher education are associated with better overall diabetes outcome two years later.

**Table 4:** Multivariable influences on outcome „T4 composite score [HbA1c/distress/QoL]”*

Predictor	Wald’s χ²	df	p	Odds ratio	95% confidence interval
Shared HbA1c goal (vs. non-shared HbA1c goal)	1.365	1	0.243	0.950	0.870–1.036
Women (vs. men)	0.340	1	0.560	1.029	0.934–1.135
Being without partner (vs. with partner)	2.958	1	0.085	1.092	0.988–1.208
Education > 8 years (vs. ≤8 years)	5.644	1	0.018	0.895	0.817–0.981
Insulin (vs. No insulin)	4.680	1	0.031	1.121	1.011–1.242
Composite score at T0	126.865	1	0.000	1.660	1.520–1.813
Age	4.047	1	0.044	0.995	0.991-1.000

Generalized Estimating Equation with cluster effect “GP practice”, normal, identity link

*composite of HbA1c/PAID/reversed EQ-5D, higher composite score = worse outcome T0 baseline, T4 two years after baseline

## Discussion

### Summary of findings

In this study, we investigated whether T2DM patients with a shared versus non-shared HbA1c target, achieved their glycemic goals. In this sample of T2DM patients with poor glycemic control GPs report a mean HbA1c goal of 7.46%. After two years we could not find statistical differences in the level of glycemic goal achievement between those with shared HbA1c goal setting versus those with non-shared goal setting. However, living with a partner had a positive impact on overall HbA1c goal achievement.

We found significant differences in the measured HbA1c values between the groups shared vs. non-shared treatment goal. Patients with a shared HbA1c goal show slightly lower HbA1c at baseline and T4. Furthermore, it could be shown that a stricter HbA1c goal was defined when a goal has been shared. One explanation for this could be that patients involved in SDM have higher knowledge and risk assessment about T2DM [38].

### Interpretation in the context of existing literature

The results of our study showed that a shared goal setting with T2DM patients had no significant impact on glycemic goal achievement. This prompted the question what impact the involvement of the patient in the decision-making process in primary care has on improving patient-relevant outcomes.

Kashaf et al. [38] conducted a systematic review to examine the impact of SDM on diabetes care outcomes and concluded that there is little evidence of a correlation between SDM and glycemic control. A total of nine included studies investigated the relation between SDM and HbA1c, only two showed a positive association. However, in most of these studies, individual treatment goals were not set [39–42].

Lafata et al. 2013 concluded that shared goal setting may not directly but indirectly lead to better glycemic control by increasing patients’ perceived self-management skills and improving the patient-physician relationship [43]. Shay et al. examined, among others the relationship between SDM and clinical patient outcomes and found no significant correlation either. But other important findings about the assessment of the SDM were obtained: (1) most of the studies measured SDM via patient self-report. However, items in these instruments do not measure what exactly prompted patients to indicate that the decision was shared; (2) unvalidated instruments have often been used to measure the relationship between patient-perceived decision making and health outcomes [44].

Another possible explanation why a shared goal setting does not lead to better glycemic goal achievement, may be due to the fact that lowering blood glucose levels is not a top priority for T2DM patients. Thus, Buhse et al. showed, that T2DM patients prioritized blood pressure control rather than intensive blood glucose control [45].

These findings suggest that the effectiveness of a shared goal setting on patient-related clinical outcomes within the process of SDM has not been fully captured yet and that broader conceptualization and measurement of SDM should be applied in future research [46].

Research has shown that SDM is not well operationalized in routine clinical practice. In a systematic review Gärtner et al. examined the quality of instruments used to assess the process of SDM. The results have shown that there is an overall lack of evidence on the quality of the measurements [47, 48]. Future studies should investigate how SDM can be integrated into routine clinical practice and which instruments are appropriate to measure SDM. National guidelines for the treatment of type 2 diabetes mellitus, provide recommendations for the implementation of SDM with a special focus on individual treatment goals, patient preferences, and treatment options in case of non-achievement of individual therapy goals [17]. These recommendations should be considered in future studies. Taking these aspects into account, it would subsequently have to be investigated whether a qualitatively good implemented SDM is associated with an improvement in HbA1c and other health related outcomes.

From our study, we also found that patients living with another person were more likely to reach their HbA1c target than patients living alone. This outcome is contrary to that of Stopford et al. 2013 who conducted a systematic review aiming to analyse the association between social support and glycemic control in T2DM. The results indicate that the marital status or living with a partner may be associated with worse glycemic control, but the evidence was limited. However, family support was found to have a beneficial effect on glycemic control [49].

Generally, there seems to be a consensus that the support of informal health supporters play an important role in managing and controlling diabetes [50]. Among other things, it was found, that perceived autonomy support from family and friends is associated with lower diabetes distress, greater diabetes management self-efficacy, and better self-monitoring of blood glucose [51]. As a hypothesis, GPs may have actively involved partners in diabetes management, decision making or in HbA1c goal achievement.

### Strengths and limitations

One of the key strengths of the DEBATE trial is its long time of follow-up with a total period of 24 months. The follow-up period of previous diabetes management studies was often less than two years [52]. Furthermore, our study included a patient population that used to be hard to reach. Despite the comparatively older, often multimorbid diabetes population, we were able to keep the dropout rate stable and within the limits of previous estimations.

The results of this study should be considered in the context of several important limitations. One is the subjective data collection of individualized treatment goals. GPs have been asked once at baseline (via questionnaire), whether they had defined a target value together with the patient. No research was done on how the GPs involved the patient in the decision-making process. No standardized procedure was given, which makes comparison between groups difficult. HbA1c target values should be closely coordinated with the patient and adjusted as needed [17]. However, it was not captured whether the treatment goal was adjusted in the further course of the study. Further research should have this in mind.

Our analysis has shown a decline of measured HbA1c levels in both groups (shared vs. non-shared) over two years. However, the clinical relevance is low. It remains unclear to what extent the decline might be ‘regression towards the mean’.

Furthermore, the potential impact of selection bias remains unclear. It can be assumed that mainly motivated and interested GPs and patients participated in the study. We cannot exclude that mainly GPs, who were already practicing SDM were included in the study. This might have diminished the effects of the DEBATE intervention as well as the group differences presented in this article. It also might have affected the external validity of the study. As no information had been collected on GPs and patients not participating in the study, the extent of a selection bias cannot be estimated.

The results of our study show, that regardless of how the HbA1c target value was set (shared or not), HbA1c levels among both groups improved. This may be due to natural reductions in patients, who were selected for their maximum HbA1c. Another reason could be the occurrence of the Hawthorne effect [52]. Even if GPs stated that they did not set the HbA1c target together with the patient, they may have involved the patient in the treatment process differently than usual due to the participation in the study. Setting HbA1c targets in a shared vs. non-shared way seems not to have an impact on HbA1c goal achievement and overall diabetes outcomes. However, in this secondary analysis we only marginally explain, which other factors might have an impact. There are many other factors (e.g., disease duration, co-morbidities, participation in DMPs) that may have influenced outcomes and were not considered in our analysis [8]. Especially, participation in DMPs and Diabetes Self-Management Education programs are important variables with a positive impact on quality of care and HbA1c target achievement [53, 54].

Furthermore, we did not examine routine GP patient care. The GPs were free in their decision to change the medication. The effect of treatment escalation on the achievement of shared vs. non-shared HbA1c goals was not investigated. However, the main analysis of the DEBATE trial shows no effect of changes in medication on HbA1c levels [26].

### Clinical impact and future research

The results of this study did not show a significant association between shared goal setting, targeting on HbA1c levels and goal achievement.

For the patient, the HbA1c value is an abstraction with many implications that are difficult to judge or even understand as a lived experience. A previous study has shown, that poorly controlled diabetes mellitus patients perceive their diabetes to a greater extent as a restriction of their daily life, independent of factors such as disease duration, comorbidities, and socio-demographic variables [19]. In practice, realistic goals should be defined that are tailored to the patient’s life situation and need.

SDM is an interaction process with the aim of reaching a jointly responsible agreement on the basis of shared information [55]. This should be developed on base of a truthful and understandable explanation from the GP trying not to influence by shaping the information in a specific direction. At the end the patient is the deciding person who has to be respected regardless of the decision made.

Future studies should focus on how SDM is implemented, what other goals are shared between GPs and patients, and the extent to which these affect the care of patients with diabetes.

## Conclusions

The secondary analyses of the DEBATE trial were not able to show that shared goal setting targeting on HbA1c-levels of patients with poorly controlled T2DM leads to improved goal achievement. Regardless of shared vs. non-shared HbA1c goal, all patients were able to lower their HbA1c-levels.

## Electronic supplementary material

Below is the link to the electronic supplementary material.

Additional file 1: Diagram flow chart patients

Additional file 2: Sensitivity analyses

## Data Availability

The datasets generated and analysed during the current study are available from the corresponding author on reasonable request.

## Declarations

### Protocol

Drewelow E, Wollny A, Pentzek M, et al. Improvement of primary health care of patients with poorly regulated diabetes mellitus type 2 using shared decision-making - the DEBATE trial. *BMC Fam Pract* 2012;13:88.

### Participating investigators

Sara Santos, Eva Drewelow, Attila Altiner, Christian Helbig, Christin Löffler, Anne Daubmann, Karl Wegscheider, Heinz-Harald Abholz, Susanne Löscher, Michael Pentzek, Stefan Wilm, Anja Wollny.

## Abbreviations

GP: General Practitioner
T2DM: Diabetes mellitus type 2
SDM: Shared Decision-Making
DMP: Disease Management Program
