# Supplementary material for: HbA1c as a shared treatment goal in type 2 diabetes? A secondary analysis of the DEBATE trial
Source: BMC Prim Care. 2023 May 13;24:115. doi: 10.1186/s12875-023-02067-9 (PMC10182591; doi:10.1186/s12875-023-02067-9)
Supplement: Supplementary file 1 — Additional file 1: Diagram flow chart patients [file 12875_2023_2067_MOESM1_ESM.pdf]

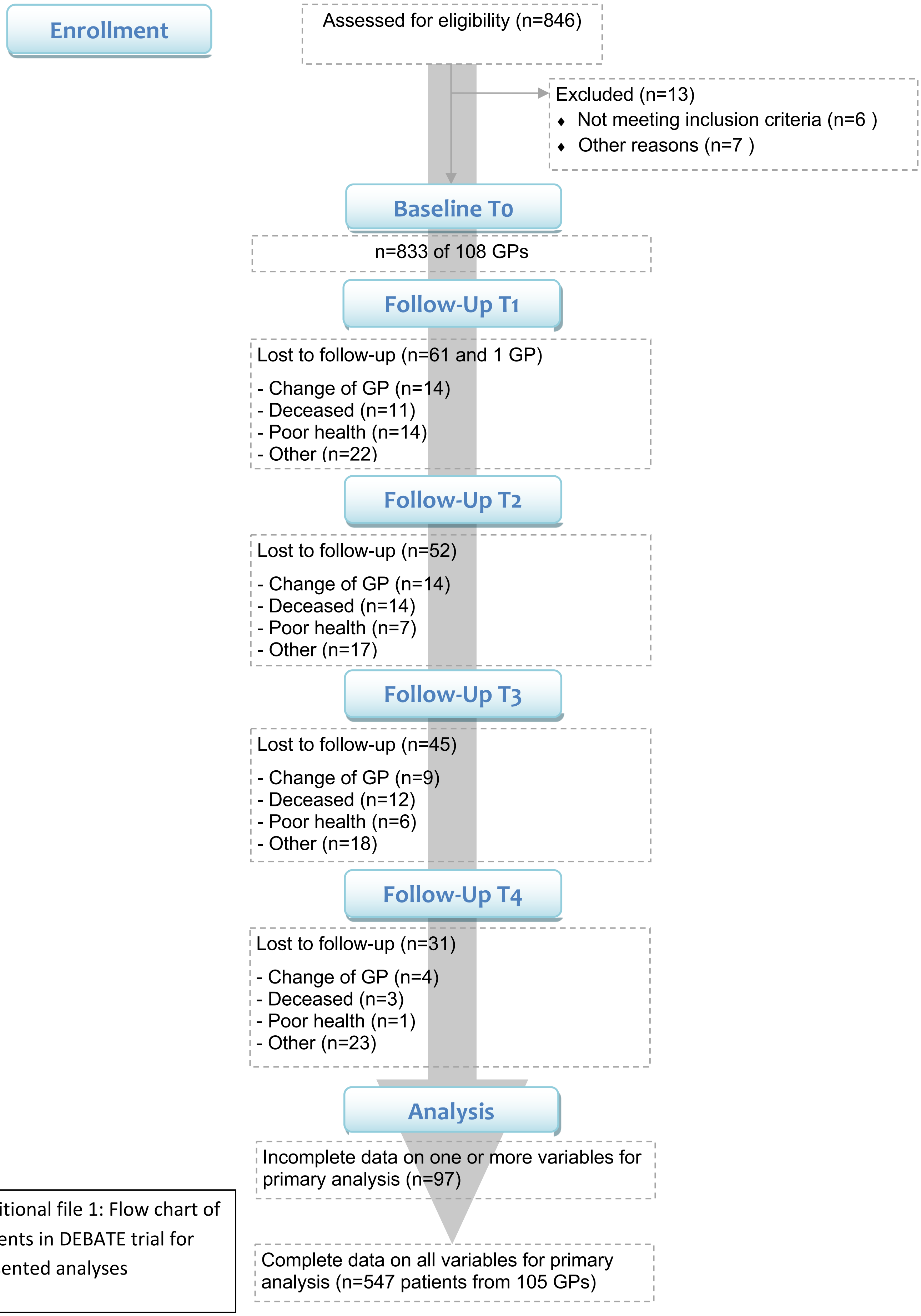

Additional file 1: Flow chart of patients in DEBATE trial for presented analyses
