# Supplementary material for: HbA1c as a shared treatment goal in type 2 diabetes? A secondary analysis of the DEBATE trial
Source: BMC Prim Care. 2023 May 13;24:115. doi: 10.1186/s12875-023-02067-9 (PMC10182591; doi:10.1186/s12875-023-02067-9)
Supplement: Supplementary file 2 — Additional file 2: Sensitivity analyses [file 12875_2023_2067_MOESM2_ESM.pdf]

Additional file 2 - Sensitivity analyses

(1) Including the intervention (control group vs. intervention group) as an additional predictor variable

	Wald's χ^2	df	p	Odds ratio	95% confidence interval	
Shared HbA1c goal (vs. non-shared HbA1c goal)	.073	1	.787	.951	.663	1.366
Women (vs. men)	.048	1	.827	1.039	.738	1.463
Being without partner (vs. with partner)	8.613	1	.003	1.877	1.233	2.857
Education >8 years (vs. ≤8 years)	1.650	1	.199	1.301	.871	1.945
Insulin (vs. no insulin)	4.044	1	.044	1.481	1.010	2.171
HbA1c value T0	10.675	1	.001	1.385	1.139	1.683
Age	1.243	1	.265	.990	.973	1.007
Control group (vs. intervention group)	1.944	1	.163	1.340	.888	2.020

Generalized Estimating Equation with cluster effect "GP practice", binomial, logit link
*higher outcome = higher risk of missing the goal (measured HbA1c at T4 is >0.5 above goal)

(2) A metric criterion (difference between measured HbA1c at T4 and HbA1c goal) instead of a dichotomous criterion (missed vs. achieved go

	Wald's χ^2	df	p	Odds ratio	95% confidence interval	
Shared HbA1c goal (vs. non-shared HbA1c goal)	.357	1	.550	.923	.709	1.201
Women (vs. men)	1.000	1	.317	.887	.701	1.122
Being without partner (vs. with partner)	12.519	1	.000	1.633	1.245	2.143
Education >8 years (vs. ≤8 years)	.175	1	.676	.951	.751	1.204
Insulin (vs. no insulin)	5.639	1	.018	1.384	1.058	1.809
HbA1c value T0	14.493	1	.000	1.302	1.137	1.492
Age	3.812	1	.051	.987	.975	1.000

Generalized Estimating Equation with cluster effect "GP practice", normal, identity link
*outcome = difference between measured HbA1c at T4 and HbA1c goal

(3a) Using outcome criteria (missed/achieved goal) from T2 (1 year after baseline) instead of T4

	Wald's χ^2	df	p	Odds ratio	95% confidence interval	
Shared HbA1c goal (vs. non-shared HbA1c goal)	1.043	1	.307	.831	.583	1.185
Women (vs. men)	1.216	1	.270	1.204	.866	1.675
Being without partner (vs. with partner)	6.231	1	.013	1.742	1.127	2.694
Education >8 years (vs. ≤8 years)	.090	1	.764	.945	.651	1.371
Insulin (vs. no insulin)	11.206	1	.001	2.038	1.343	3.093
HbA1c value T0	3.461	1	.063	1.212	.990	1.483
Age	7.867	1	.005	.971	.951	.991

Generalized Estimating Equation with cluster effect "GP practice", binomial, logit link
*higher outcome = higher risk of missing the goal (measured HbA1c at T2 is >0.5 above goal)

(3b) Using outcome criteria (difference measured – aspired HbA1c) from T2 (1 year after baseline) instead of T4

	Wald's χ^2	df	p	Odds ratio	95% confidence interval	
Shared HbA1c goal (vs. non-shared HbA1c goal)	.270	1	.603	.931	.710	1.220
Women (vs. men)	.175	1	.676	1.046	.848	1.288
Being without partner (vs. with partner)	11.236	1	.001	1.486	1.179	1.873
Education >8 years (vs. ≤8 years)	.001	1	.971	.996	.801	1.239
Insulin (vs. no insulin)	6.612	1	.010	1.425	1.088	1.867
HbA1c value T0	11.105	1	.001	1.244	1.094	1.414
Age	10.155	1	.001	.980	.969	.992

Generalized Estimating Equation with cluster effect "GP practice", normal, identity link
*outcome = difference between measured HbA1c at T2 and HbA1c goal

(4a) Including the shared-decision making score (SDM-Q-9) as an additional predictor variable (as a main effect)

	Wald's χ²	df	p	Odds ratio	95% confidence interval	
Shared HbA1c goal (vs. non-shared HbA1c goal)	.092	1	.761	.943	.648	1.373
Women (vs. men)	.188	1	.665	1.079	.764	1.525
Being without partner (vs. with partner)	8.170	1	.004	1.830	1.209	2.769
Education >8 years (vs. ≤8 years)	1.751	1	.186	1.317	.876	1.981
Insulin (vs. no insulin)	3.694	1	.055	1.464	.992	2.160
HbA1c value T0	10.225	1	.001	1.373	1.131	1.668
Age	1.194	1	.275	.990	.972	1.008
SDM-Q-9_sum at T0	.472	1	.492	1.004	.992	1.017

Generalized Estimating Equation with cluster effect "GP practice", binomial, logit link
*higher outcome = higher risk of missing the goal (measured HbA1c at T4 is >0.5 above goal)

(4b) Including the shared-decision making score (SDM-Q-9) as an additional predictor variable (as an interaction term SDM x shared/non sh

	Wald's χ²	df	p	Odds ratio	95% confidence interval	
Women (vs. men)	.116	1	.734	1.062	.752	1.498
Being without partner (vs. with partner)	8.301	1	.004	1.837	1.215	2.779
Education >8 years (vs. ≤8 years)	1.612	1	.204	1.306	.865	1.970
Insulin (vs. no insulin)	4.030	1	.045	1.494	1.010	2.211
HbA1c value T0	10.614	1	.001	1.387	1.139	1.689
Age	1.109	1	.292	.990	.972	1.008
Shared HbA1c goal * SDM-Q-9_sum at T0	1.095	1	.295	1.007	.994	1.020
Non-shared HbA1c goal * SDM-Q-9_sum at T0	.038	1	.845	1.002	.986	1.017

Generalized Estimating Equation with cluster effect "GP practice", binomial, logit link
*higher outcome = higher risk of missing the goal (measured HbA1c at T4 is >0.5 above goal)
